# The Effect of Smoking Behavior on Alveolar Bone Marrow Mesenchymal Stem Cells of Clinical Implant Patient

**DOI:** 10.1155/2018/7672695

**Published:** 2018-11-21

**Authors:** Xicong Zhao, Bin Zhu, Yan Duan, Xin. Wang, Dehua Li

**Affiliations:** ^1^State Key Laboratory of Military Stomatology and National Clinical Research Center for Oral Diseases and Shaanxi Key Laboratory of Oral Diseases, Department of Oral Implants, Fourth Military Medical University, Xi' an, Shaanxi, China; ^2^Department of Stomatology, Affiliated Heping Hospital, Changzhi Medical College, Changzhi, Shanxi, China; ^3^Department of Stomatology, PLA Xizang Military Region General Hospital, Lhasa, Tibet, China

## Abstract

**Objective:**

The hazardous effects of smoking on the alveolar bone healing after implant surgery and nicotine on the biofunction of human alveolar bone marrow mesenchymal stem cells (hABMMSCs) were reported. There was little direct evidence regarding the specific detrimental effects of the smoking on hABMMSCs. The aim of this study was to test the influence of smoking behavior on hABMMSCs and the osseointegration situation after implant surgery.

**Methods:**

hABMMSCs from 6 dental implant patients randomly (3 smokers and 3 nonsmokers) were compared. The cell viability, colony forming unit, and cell cycle were performed to assay proliferation capacity. The Oil Red O staining, Alizarin Red staining, alkaline phosphatase staining and activity, adipogenic and osteogenic gene expressions in vitro, and bone formation ectopically in vivo were performed under proper inductions, respectively, to assay multilineage differentiation. Besides the implant stability quotient and marginal bone loss were checked in both groups.

**Results:**

Smoking hABMMSCs showed lower proliferation in vitro and poorer bone regeneration capacity in vivo. Moreover, smokers performed worse on bone healing after implant surgery.

**Conclusions:**

Our results suggested smoking had the detrimental genetic effect on proliferation and osteogenesis of hABMMSCs and the decreased biofunction of hABMMSCs was positively related with bone healing.

**Clinical Significance:**

The present study provided direct evidence about hazardous effects of smoking behavior on hABMMSCs. Smoking decreased the osteogenesis and proliferation of hABMMSCs in vivo and in vitro, and smoking was positively related with osseointegration reduction. Prevention of smoking behavior may promote biofunction of hABMMSCs and successful rate of dental implant.

## 1. Introduction

Dental implant has been the top choice for dentition defect over past decade. The success rate of implant was promoted with the development of surgery skills, Ti-surface treatment, and late maintenance. However, failure would happen sometimes and the smoking behavior was one of the definite factors according to the recent studies [1, 2]. Smoking behavior was a clear predisposing factor for many diseases, including lung cancer, cardiovascular diseases, osteoporosis, oral cancer, and periodontal diseases [3, 4]. Clinical researches showed smokers possessed a higher failure rate of dental implant than nonsmokers [5, 6]. Moreover, a greater detrimental effect on the successfully integrated implants was reported [7, 8]. Meta-analysis also confirmed that the failure rate of smokers was significantly higher [1]. Based on laboratory evidence, the negative effects of smoking behavior on the postoperative bone healing of titanium implants were demonstrated in rats [9, 10]. Accordingly smoking behavior proved to have a definite negative effect on the success rate of implants.

Human alveolar bone marrow mesenchymal stem cells (hABMMSCs) possessing multipotential differentiation participated in the repair and regeneration of jawbone and periodontal tissue [11–13]. Recently, many studies focused on the harmful consequence of smoking on the dental implant and nicotine was demonstrated to be harmful to hABMMSCs from nonsmokers [10]. However, there was little direct evidence that smoking behavior affected biofunction of hABMMSCs. Therefore, we investigated biology behavior difference of hABMMSCs between smoking and nonsmoking patient. Moreover, the implant stability quotient (ISQ) and marginal bone loss (MBL) were checked in both groups. The effect of smoking behavior on hABMMSCs and periodontal situation postoperatively would be researched.

## 2. Materials and Methods

### 2.1. Study Subjects

Alveolar bone marrow aspirates were collected from drill holes in the alveolar bone of 6 dental implant patients (3 smokers and 3 nonsmokers) randomly. All samples were collected at the School of the Stomatology of the Fourth Military Medical University. The subjects in the study had no history of systemic disease. The study was approved by the Fourth Military Medical University Ethics Committee, and informed consent was obtained from the patients.

### 2.2. Isolation and Culture of hABMMSCs

The isolation and culture of hABMMSCs from smoking and nonsmoking patient were as previously described [13]. Multiple colony-derived hABMMSCs at 2-4 passages were used in our experiments.

### 2.3. Flow Cytometry (FCM) Analysis

#### 2.3.1. Cell Surface Markers

To identify the s-hABMMSCs and n-hABMMSCs phenotype, cells at the third passage were trypsinized and centrifuged. Approximately 5 × 10^5^ cells were incubated with phycoerythrin (PE)- or fluorescein isothiocyanate (FITC)-conjugated monoclonal antibodies for human CD34 (Chemicon), CD44, CD90, CD45, CD31, CD105, CD29 (eBioscience, San Diego, CA), STRO-1, and CD146 (R&D Systems) according to the manufacturer's protocol. The secondary reagents included goat anti-mouse and goat anti-rat IgG-FITC (Santa Cruz Biotechnology). Cells were analyzed on FCM (Becton-Dickinson, San Jose, CA, USA), and the data were analyzed with Cell Quest software.

### 2.4. Cell Cycle Analysis

The s-hABMMSCs and n-hABMMSCs were fixed with 70% alcohol overnight at 4°C. The fixed cells were washed twice with PBS and stained with 100 mg/mL propidium iodide (PI) (Sigma-Aldrich Corp) at 4°C for 30 minutes. The PI-elicited fluorescence of individual cells was measured using FCM (Beckman Coulter, USA). At least 5 × 10^5^ cells were analyzed for each sample. The amounts of cells residing in the G0/G1 phase, S phase, and G2/M phase were determined.

### 2.5. Colony Forming Unit (CFU) Assay

Single-cell suspensions (1 × 10^3^) within *α*-MEM (10% FBS) were seeded in 10 cm diameter culture dishes (Corning, Lowell, MA, USA). After 14 days of cultivation, cultures were fixed with 4% paraformaldehyde and stained with 0.1% toluidine blue. Aggregates containing 50 or more cells were counted as colonies under the microscope. The numbers of colonies per well were counted. The experiment repeated at least three times.

### 2.6. 3-(4, 5-Dimethylthiazol-2yl)-2, 5-Diphenyltetrazolium Bromide (MTT) Assay

Cells were plated in a 96-well culture plate at a density of 1.5 × 10^3^ cells per well in *α*-MEM with 10% FBS. At the end of the test time points (1 to 8 each day), cell viability of s-hABMMSCs and n-hABMMSCs was assessed using methyl tetrazolium methods (MTT; Sigma) according to the manufacturer's instructions [11]. The optical density (OD) values were determined with a multiplate reader (ELx800, BioTek Instruments Inc., USA) at a wavelength of 490 nm. This test was repeated 3 times.

### 2.7. Osteogenic Differentiation

The osteogenic induction medium containing *α*-MEM with 10% FBS, 0.1 *μ*M dexamethasone, 10 mM *β*-glycerol phosphate (Sigma, USA), and 50 mg/L ascorbic acid was used to the s-hABMMSCs and n-hABMMSCs of passage 3 for four weeks. The Alizarin red-staining was performed as previously [11]. The stained matrix was routinely observed and photographed under a phase-contrast inverted microscope (Olympus Optical, Tokyo, Japan). The Alizarin red-stained area was semi-quantitatively measured by Leica Q-Win image analysis system (Leica, Germany).

### 2.8. Alkaline Phosphatase (ALP) Activity and Staining Assay

Single-cell suspensions of s-hABMMSCs and n-hABMMSCs were seeded at a density of 2 × 10^5^ cells into 75 cm^2^ culture dish. And after 14 days of culture with osteogenic induction, ALP staining was determined with the BCIP/NBT Alkaline Phosphatase Color Development Kit (Beyotime Co., Shanghai, China), as previously described [11].

### 2.9. Adipogenic Differentiation

The adipogenic induction medium consisting of *α*-MEM with 10% FBS, 0.25 *μ*M dexamethasone, 100 *μ*M indomethacin (Sigma, USA), 0.5 mM 3-isobutyl methylxanthine (Sigma, USA), and 10 mg/L insulin was used for two weeks. The lipid droplets were stained with Oil-red O (Sigma, USA). To obtain quantitative data, 1 ml of isopropyl alcohol was added to the stained culture dish. After 5 minutes, the absorbance of the extract was assayed by a spectrophotometer at 520 nm after dilution to a linear range.

### 2.10. Total RNA Extraction and Quantitative Real-Time Reverse-Transcriptase Polymerase Chain Reaction (qRT-PCR)

The qRT-PCR performance was as previously described [14]. The primers for the target genes were listed in Supplementary Table 1. The expression levels of the target genes were normalized to that of the housekeeping gene *β*-actin.

### 2.11. Scaffold Materials and Cell Aggregate (CA) Preparation and Observation

The sterilized hydroxyapatite-polylactic acid (HA-PLA) (5 × 5 × 3 mm^3^) (Research and Development Center for Tissue Engineering, Fourth Military Medical University, Xi'an, China) was used as scaffold material in our study.

hABMMSCs from 3 nonsmokers and 3 smokers at passage 3 were, respectively, cultured to CAs as previously described [11]. Each HA-PLA scaffold block was coated with 3 layers of hABMMSCs CAs from one patient successively to construct one transplant (Figure 5(a)), and 3 transplants from one patient were cultured with osteogenic induction for 3 days. And then the transplants were fixed and observed by scanning electron microscope (SEM) (Hitachi, S-4800. Japan).

### 2.12. Nude Mice Ectopic Transplantation

All the animal procedures complied with the guidelines provided by the Animal Care Committee of the Fourth Military Medical University. The transplants from nonsmokers and smokers were subcutaneously transplanted into different sides of subcutaneous pockets separately of nine 8-week-old nude mice (BALB/c-nu; FMMU Medical Laboratory Animal Center, Xi'an, China), respectively. All nude mice were injected with 0.1 *μ*g/g mebumalnatrium for anesthesia. After eight weeks of the transplantation, all fifteen nude mice were euthanized, and all 18 transplants were removed for analysis.

### 2.13. Hematoxylin and Eosin (H&E), Masson Trichrome, and Immunohistochemical Staining

The transplants were fixed in 4% paraformaldehyde for 24 hours, paraffin-embedded, longitudinally sectioned, and stained with H&E and Masson trichrome staining, respectively, as previously described [14]. Other sections were incubated with primary antibodies following anti-Col-I (1:200, Abcam, British). The phosphate-buffered solution (PBS) was used for the negative controls instead of the primary antibodies. Biotinylated secondary antibodies (1:1000) were purchased from Dako (Dako, USA). The staining sections were observed with a light microscope (Nikon, Japan). The positive stained mineralized area was quantitatively measured by Leica Q-Win image analysis system (Leica, Germany).

### 2.14. Implant Stability Quotient (ISQ) and Periodontal Situation after Implant Placement

The implant surgery was performed according to standard protocols. All 10 Straumann dental implants were placed for 6 patients, and each implant per patient was randomly selected.

ISQ was detected by resonance frequency analysis after implant insertion immediately and at 1, 2, 3, 4, 6, 8, and 12 weeks postoperatively as previously described [15]. Values were recorded using an Osstellt™ Mentor (Integration Diagnostics AB, Goteborg, Sweden).

At 6 and 12 months after implant restoration, peri-implant soft tissue condition including modified plaque index (mPLI), modified sulcus bleeding index (mSBI), and probing depth (PD), and marginal bone level (MBL) was checked by the same clinician as previously described [7, 16].

mPLI and mSBI was measured at four aspects around the implant. They were assessed at four aspects of each implant. The average of the four measured values was used as the value that implant.

PD was assessed at four aspects of each implant in millimeters. The average of the four measured values was used as the PD for that implant.

MBL was measured by examining the panoramic radiographs and comparing them with the initial measured MBL (abutment placement). The average of the two examiners' results was used as the finial MBL.

### 2.15. Statistical Analysis

All data were expressed as mean ± SD and analyzed by two-tailed unpaired Student's t-test using SPSS software (version 12.0, SPSS, Chicago, IL). P-values <0.05 were considered statistically significant.

## 3. Result

### 3.1. Condition of Experiment Subjects

We obtained alveolar bone marrow samples from 6 dental implant patients (3 smokers and 3 nonsmokers). As Supplementary Table 2 shows, all 6 implant sites were in the posterior maxilla area. The bone morphology for all 6 implants was rated as Type II or Type III. And smoking patient had a heavy smoke history.

### 3.2. Morphological Feature and Proliferative Potential

**Figure 1:**
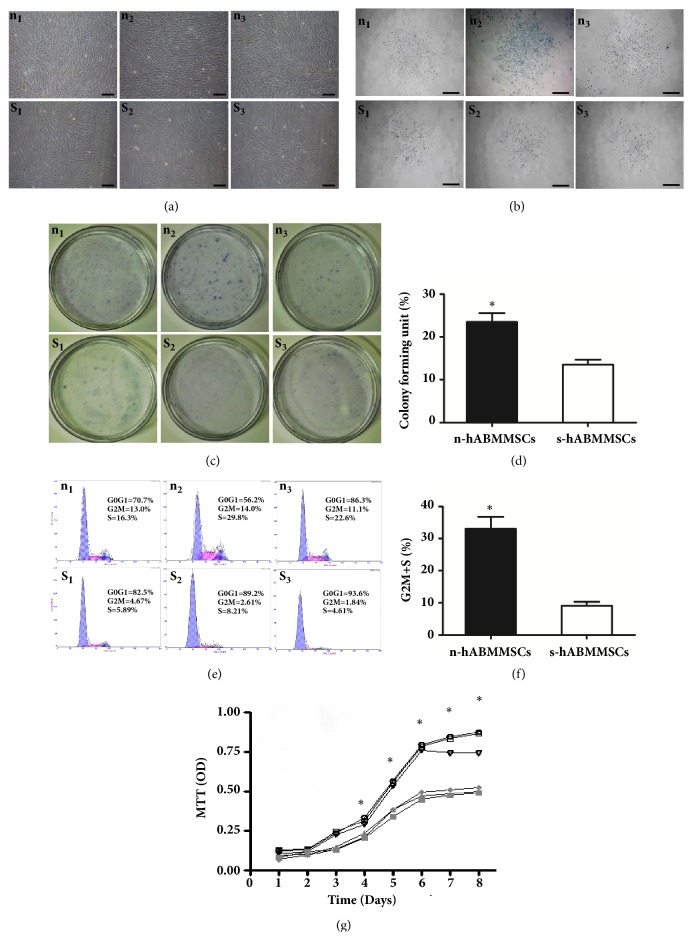


The s-hABMMSCs and n-hABMMSCs all showed a typical fibroblast-like spindle appearance (Figure 1(a)) and had the ability to form adherent clonogenic cell clusters (Figure 1(b)). Meanwhile, there was a statistically significant difference of CFU between n-hABMMSCs (22.8±2.02%) and s-hABMMSCs (11.2±0.95%) (Figures 1(c) and 1(d)).

The results of cell cycle analysis further confirmed that the proliferation ability of n-hABMMSCs was higher than that of s-hABMMSCs, which was shown by the fact that n-hABMMSCs exhibited significantly higher percentages of cells in G2+S phases than s-hABMMSCs (Figures 1(e) and 1(f)). The MTT test for consecutive 8 days indicated that n-hABMMSCs possessed a higher proliferative potential than s-hABMMSCs and the difference became significant from day 4 (Figure 1(g)).

### 3.3. Characterization of Epitope Profile

**Figure 2:**
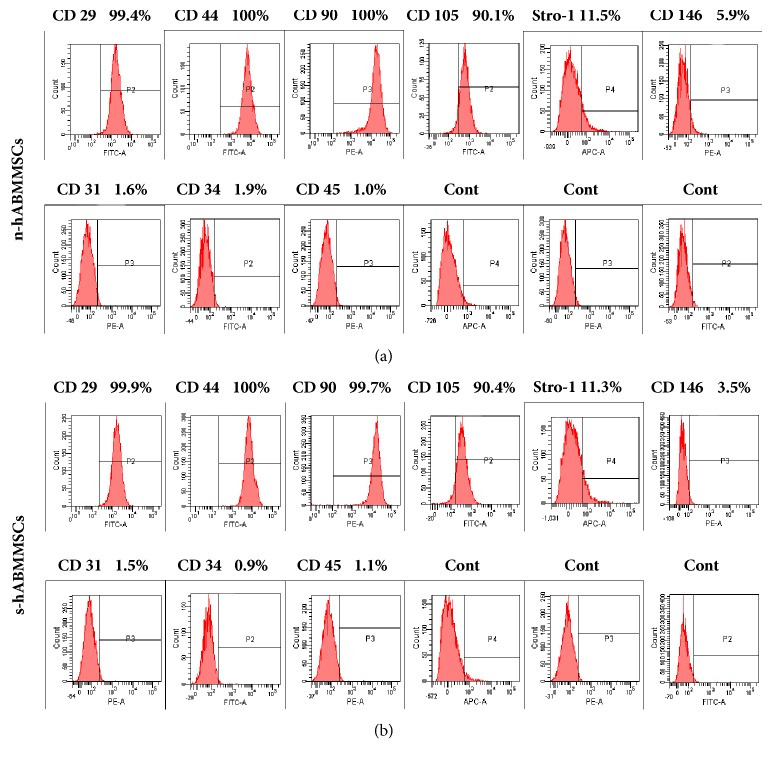


Fluorescence-activated cell sorting analysis for epitope profile showed that s-hABMMSCs (Figure 2(b)) were similar to n-hABMMSCs (Figure 2(a)), expressed CD29, CD44, CD90, CD105, CD146, and STRO-1, the putative mesenchymal stem cell markers at the high level, respectively, and expressed negatively hematopoietic lineage markers, including CD34 and CD45, and platelet endothelial cell makers CD31.

### 3.4. In Vitro Osteogenesis Ability of s-hABMMSCs and n-hABMMSCs

**Figure 3:**
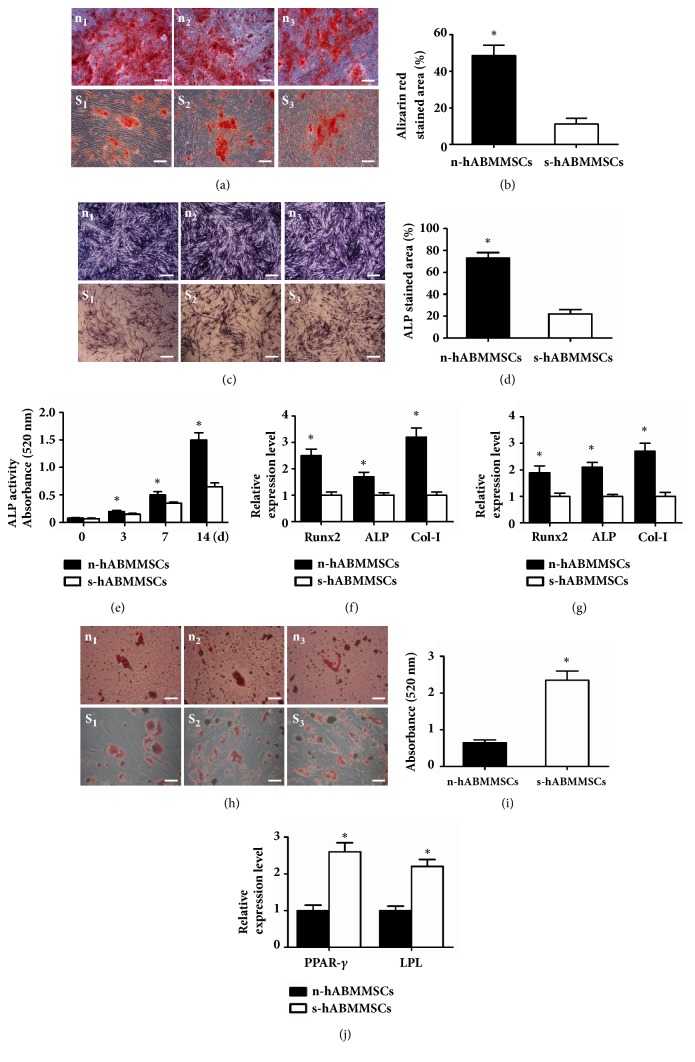


After osteogenic induction for 4 weeks, mineralized extracellular matrix (ECM) could be shown with Alizarin Red staining in both cell populations (Figure 3(a)). Quantification of the Alizarin Red–positive area showed that the extracellular ECM per microscopical field was 48.71% in n-hABMMSCs and 11.43% in s-hABMMSCs (P< 0.05) (Figure 3(b)). After osteogenic induction for 1 week, ALP staining was also performed (Figure 3(c)). Quantification of the ALP stained area showed that n-hABMMSCs accessed higher ALP staining area (76.8%) than s-hABMMSCs (22.4%) (P< 0.05) (Figure 3(d)). ALP activity assay at different time points indicated that the ALP activity of n-hABMMSCs was much higher than that of s-hABMMSCs (P< 0.05) (Figure 3(e)).

Further research on mRNA relative intensities suggested that n-hABMMSCs expressed mineralization markers (ALP, Col-I, and Runx2) at higher levels in comparison with s-hABMMSCs after osteogenic induction for 1 and 2 weeks (P< 0.05) (Figures 3(f) and 3(g)).

### 3.5. In Vitro Adipogenesis Ability of s-hABMMSCs and n-hABMMSCs

Both cell populations formed Oil Red O positive lipid clusters after 3 weeks of adipogenic induction (Figure 3(h)); and quantification of the Oil Red O stained lipid clusters showed absorbance of n-hABMMSCs (0.81) was lower than that of s-hABMMSCs (2.27) (P< 0.05) (Figure 3(i)). Moreover, n-hABMMSCs exhibited higher level of adipogenic relative gene expression peroxisome proliferation activated receptor-*γ* (PPAR-*γ*) and lipoprotein lipase (LPL), in comparison with s-hABMMSCs after osteogenic induction for 1 week (P< 0.05) (Figure 3(j)).

### 3.6. In Vivo Osteogenesis Ability of s-hABMMSCs and n-hABMMSCs

Three layers CAs of n-hABMMSCs or s-hABMMSCs with HA-PLA scaffold were cultured with osteogenic induction for 3 days. The SEM pictures showed that CAs could adhere to the scaffolds well, proliferate adequately, and extend excessively on the surface of HA-PLA (Figure 5(b)).

**Figure 4:**
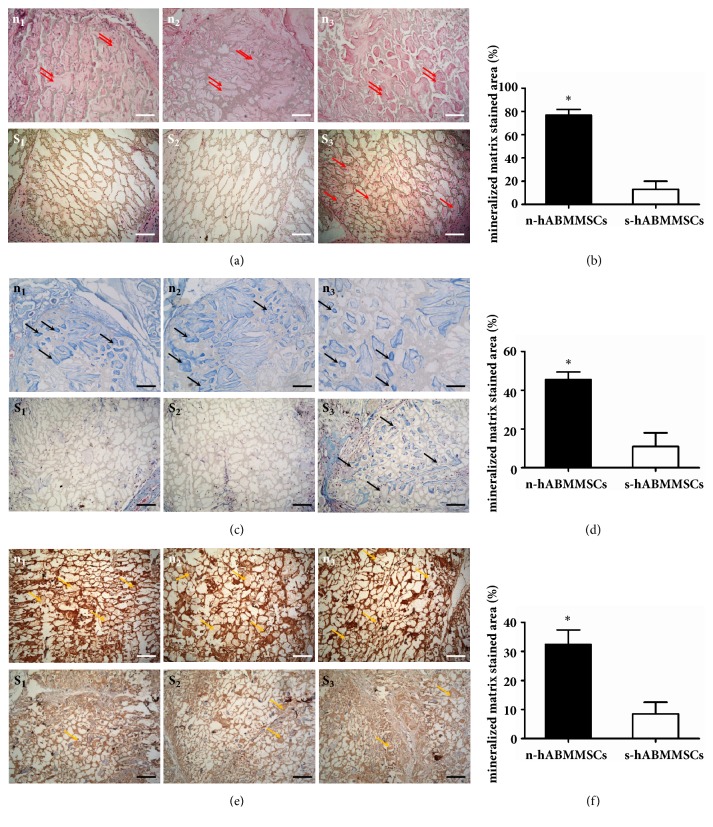


Eight weeks after transplantation in nude mice, 18 experiment specimens were harvested and examined by H&E, Masson trichrome, and immunohistochemical staining. In the n-hABMMSCs group, a large number of mineralized ECM newly formed between the HA-PLA scaffold materials. On the contrary, in the n-hABMMSCs group, seldom newly mineralized ECM was observed (Figures 4(a), 4(c), and 4(e)).

**Figure 5:**
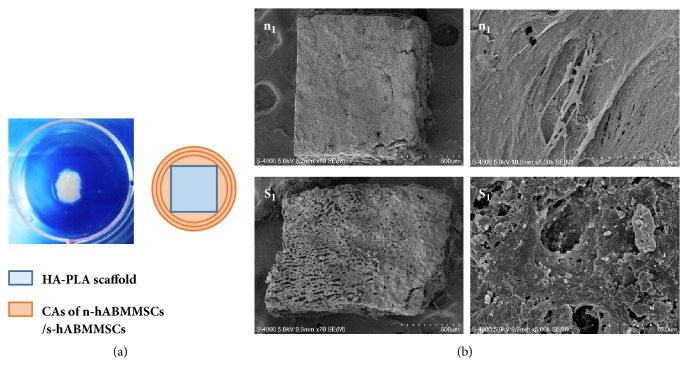


In the H&E staining, newly bone matrix was stained by eosin (red arrow) and HA-PLA material was shown in brown scaffold (Figure 5(a)). Leica Q-Win image analysis showed that n-hABMMSCs (78.1%) obtained a higher bone matrix formation in the nude mice than s-hABMMSCs on average (17.4%) (P< 0.05) (Figure 4(b)). Meanwhile the Masson trichrome and immunohistochemical staining showed the same results. The black and yellow arrows indicated newly bone matrix in Masson trichrome and immunohistochemical staining, respectively (Figures 4(c) and 4(e)). Newly bone matrix formation of n-hABMMSCs was shown to be over 3-fold higher than that of s-hABMMSCs in Masson trichrome (n-hABMMSCs with 47.3% and s-hABMMSCs with 15.3%) (Figure 4(d)) and immunohistochemical (n-hABMMSCs with 33.6% and s-hABMMSCs with 8.9%) (Figure 4(f)) staining, respectively (P< 0.05).

### 3.7. Osseointegration and Periodontal Situation after Placement

All implants from six patients achieved osseointegration without any complication. The mean ISQ value of both groups decreased for 2 weeks after implant surgery and increased steadily for the next 10 weeks. The mean ISQ value showed significant difference between nonsmoking and smoking patients from the 3rd to 6th week after implantation (P< 0.05, Table 1). In contrast, no difference was observed at the first 3 weeks nor the last 4 weeks (P> 0.05). And ISQ of nonsmoking patient increased dramatically from the 3rd to 4th week after implantation compared with smoking patient.

The MBL of smoking patient was dramatically lower than that of nonsmoking at 6 and 12 months after loading (P< 0.0001, Table 2). The mSBI and mPLI showed no significant difference between smoking and nonsmoking patient at 6 and 12 months after loading (P> 0.05, Table 3). And the PD differed significantly between groups at 12th week postoperatively (P< 0.05, Table 3).

## 4. Discussion

Smoking was associated with excessive destruction of the supporting periodontal tissues, resulting in pocket formation, bone loss, and premature tooth loss [17–19]. Among more than 4,000 chemicals present in tobacco smoke, nicotine was now recognized as a modulator of key cellular proteins and processes involved in the pathobiological effects of tobacco in nonneuronal locations [20]. Nicotine acting through the nAChRs expressed by nonneuronal cells had emerged as a candidate for the major pathogenic factor in tobacco-related morbidity [4, 21, 22].

In this study, our findings were similar to other mesenchymal stem cells. The s-hABMMSCs and n-hABMMSCs exhibit the self-renewal capacity to form clonogenic clusters and undergo extensive proliferation in vitro. Meanwhile, s-hABMMSCs and n-hABMMSCs express mesenchymal stem cell makers CD29, CD44, CD90, CD105, CD146, and STRO-1, lacking expression of hematopoietic markers CD34, CD45, and CD31. With the comparison of growth potential with s-hABMMSCs and n-hABMMSCs, n-hABMMSCs have a higher proliferation rate than those s-hABMMSCs. The n-hABMMSCs exhibited much higher osteogenic potential than s-hABMMSCs. The s-hABMMSCs exhibited much higher adipogenic potential than n-hABMMSCs. This means the patient's daily smoking habit would affect the gene expression of hABMMSCs.

Otherwise, the success rate of the endosseous implant relies primarily on the mechanism of wound healing and the ability of the alveolar bone to rebuild and secure the titanium screw within the newly formed bone [23]. Hematopoietic stem cells might have migrated from some bones to others [24], because it has been suggested that BMMSCs migrate from bone marrow to injured tissues [25]. Recently, a study using micro computerized tomography also reported that daily administration of nicotine caused alveolar bone loss and microstructure deteriorations in a dose-dependent manner [26]. In vivo studies revealed that, compared to saline solution, daily administration of nicotine might enhance the effects of periodontal bone loss [9]. Our study gave the supplementary cell-level changes occurring in the smoking patients.

Osseointegration is the formation of a direct interface between an implant and bone, without intervening soft tissue [27]. And osseointegrated implant could take loading as basis of function [28]. Implant stability quotient (ISQ) measured by RFA could reflect the interconnection between implant and bone [15]. And our findings were coincident with previous multiple longitudinal studies that the mean ISQ decreased at the beginning stage and increased steadily afterward [29, 30]. The alveolar bone resorption and reconstruction was believed to be closely related with this change [31]. Moreover, ISQ of smoking patient increased more slowly than nonsmoking patient significantly after decreasing stage. This finding verified smoking behavior affected alveolar bone reconstruction [32]. MBL was measured by two-dimensional imageological examination which reflected integration between implant and bone [33, 34]. Our results certified that smoking behavior was positively related with bone resorption around implant. However, mSBI and mPLI showed no significant difference between two groups. Insufficient data size may induce the opposite result against the previous studies [7, 16].

## 5. Conclusions

The present study suggested the heavy smoking behaviour would downregulate biofunction of human alveolar bone marrow mesenchymal stem cells, including proliferation and osteogenesis differentiation. And these were positively related to decrease of integration between alveolar bone and implant.

Our results suggested smoking had the detrimental genetic effect on proliferation and osteogenesis of hABMMSCs and the decreased biofunction of hABMMSCs was positively related with bone healing. Prevention of smoking behavior may promote biofunction of hABMMSCs and successful rate of dental implant.

## Figures and Tables

**Table 1:** ISQ of all dental implants between two groups at each time point. N1: nonsmoking patient NO.1; N2: nonsmoking patient NO.2; N3: nonsmoking patient NO.3; N: the mean value of nonsmoking patient; S1: smoking patient NO.1; S2: smoking patient NO.2; S3: smoking patient NO.3; S: the mean value of smoking patient; ns: nonsense; ISQ: implant stability quotient. P value was calculated from two-sided t-test.

Time (weeks)	N1	N2	N3	N (mean±SD)	S1	S2	S3	S (mean±SD)	P
0	79	82	85	82.00±1.732	85	78	79	80.67±2.186	0.4542 ns
1	76	77	80	77.00±1.528	81	75	74	76.66±2.667	0.5257 ns
2	67	70	72	69.67±1.453	76	72	66	71.33±1.856	0.4245 ns
3	75	77	81	77.67±3.512	77	71	69	72.33±3.333	0.0363 (*∗*)
4	78	80	78	78.67±1.453	78	73	73	74.67±2.082	0.0182 (*∗*)
6	79	82	80	80.33±1.667	80	75	77	77.33±1.856	0.0378 (*∗*)
8	80	84	80	81.33±0.881	82	75	79	78.67±1.000	0.1297 ns
12	80	85	82	82.33±0.333	83	76	80	79.67±0.881	0.1377 ns

**Table 2:** MBL of dental implants between two groups at two time points. N1: nonsmoking patient NO.1; N2: nonsmoking patient NO.2; N3: nonsmoking patient NO.3; N: the mean value of nonsmoking patient; S1: smoking patient NO.1; S2: smoking patient NO.2; S3: smoking patient NO.3; S: the mean value of smoking patient; MBL: marginal bone loss. P values were calculated from two-sided t-test.

Time (months)	N1	N2	N3	N (mean±SD)	S1	S2	S3	S (mean±SD)	P
6	0.85	0.89	0.72	0.80±0.039	1.58	1.77	1.83	1.73±0.075	<0.0001 (*∗∗∗*)
12	0.96	0.98	0.82	0.92±0.050	1.78	2.02	1.95	1.92±0.071	<0.0001 (*∗∗∗*)

**Table 3:** Periodontal situation of all dental implants between two groups at each time point. N1: nonsmoking patient NO.1; N2: nonsmoking patient NO.2; N3: nonsmoking patient NO.3; N: the mean value of nonsmoking patient; S1: smoking patient NO.1; S2: smoking patient NO.2; S3: smoking patient NO.3; S: the mean value of smoking patient; ns: nonsense; P value was calculated from two-sided t-test.

Time (months)	Item	N1	N2	N3	N (mean±SD)	S1	S2	S3	S (mean±SD)	P
6	mSBI	0	0.25	0.75	0.33±0.220	0.75	0.50	0	0.42±0.220	0.6675 ns
	mPLI	0.25	0.25	1.00	0.50±0.250	0.50	0.25	0.25	0.33±0.083	0.3349 ns
	PD (mm)	1.25	1.50	1.75	1.50±0.144	2.25	1.75	1.25	1.75±0.289	0.2508 ns

12	mSBI	0.50	1.00	1.50	1.00±0.289	1.25	1.75	1.50	1.83±0.220	0.0550 ns
	mPLI	1.00	1.50	1.75	1.25±0.250	2.50	1.50	1.75	2.00±0.144	0.0808 ns
	PD (mm)	1.75	2.50	2.75	2.33±0.300	3.50	3.25	2.50	3.08±0.300	0.0378 (*∗*)

## Data Availability

The original data of this study can be offered by the corresponding author on reasonable request under the consent of the State Key Laboratory of Military Stomatology and National Clinical Research Center for Oral Diseases and Shaanxi Key Laboratory of Oral Diseases, Fourth Military Medical University.

## Supplementary Materials

Supplementary Materials Supplementary Table 1: primer sequences. Primer sequences of osteogenesis related gene including Runx2, Col-I, and ALP were used for RT-PCR to test osteogenic differentiation. And primer sequences of adipogenesis related gene including LPL and PPAR-*γ* were used for RT-PCR to test adipogenic differentiation. Supplementary Table 2: conditions of donators of hABMMSCs. Individual information including smoking and bone situation was collected from both nonsmoking and smoking patient. Compared with nonsmoking patient, the smoking amount (more than 10 cigarettes/day) and history (longer than 10 years) of smoking patient reached the heavy smoking level.
