# Supplementary material for: The Effect of Smoking Behavior on Alveolar Bone Marrow Mesenchymal Stem Cells of Clinical Implant Patient
Source: Biomed Res Int. 2018 Nov 21;2018:7672695. doi: 10.1155/2018/7672695 (PMC6280244; doi:10.1155/2018/7672695)
Supplement: Supplementary Materials — Supplementary Table 1: primer sequences. Primer sequences of osteogenesis related gene including Runx2, Col-I, and ALP were used for RT-PCR to test osteogenic differentiation. And primer sequences of adipogenesis related gene including LPL and PPAR-γ were used for RT-PCR to test adipogenic differentiation. Supplementary Table 2: conditions of donators of hABMMSCs. Individual information including smoking and bone situation was collected from both nonsmoking and smoking patient. Compared with nonsmoking patient, the smoking amount (more than 10 cigarettes/day) and history (longer than 10 years) of smoking patient reached the heavy smoking level. [file 7672695.f1.pdf]

The effect of smoking behavior on alveolar bone marrow mesenchymal stem cells of clinical implant patient

Xicong Zhao[1,2†], Bin Zhu[1,3†], Yan Duan[1], Xin. Wang[1], Dehua Li[1]

1. State Key Laboratory of Military Stomatology &National Clinical Research Center for Oral Diseases&Shaanxi Key Laboratory of Oral Diseases, Department of Oral Implants, Fourth Military Medical University, Xi' an, Shaanxi, People' s Republic of China.

2. Department of Stomatology, Affiliated Heping Hospital, Changzhi Medical College, Changzhi, Shanxi, People' s Republic of China.

3. Department of Stomatology, PLA Xizang Military Region General Hospital, Lhasa, Tibet, People' s Republic of China.

Corresponding Author:

Prof. D. Li

Add.: Department of Oral Implant, State Key Laboratory of Military Stomatology, School of Stomatology, Fourth Military Medical University, No.145 West Changle Road, Xi' an, Shaanxi 710032, People' s Republic of China.

Tel.: +86-29-84776451

E-mail: lidehua@fmmu.edu.cn

Supplementary Table 1. Primer Sequences.

Gene	Forward	Reverse
Runx2	5’-CCCGTGGCCTTCAAGGT-3’	5’-CGTTACCCGCCATGACAGTA-3’
Col-I	5’-CCAGAAGAACTGGTACATCAGCAA-3’	5’-CGCCATACTCGAACTGGAATC-3’
ALP	5’-TAAGGACATCGCCTACCAGCTC-3’	5’-TCTTCCAGGTGTCAACGAGGT-3’
LPL	5’-AGGACCCCTGAAGACAC-3’	5’-GGCACCCAACTCTCATA-3’
PPAR-γ	5’-CAAGACAACCTGCTACAAGC-3’	5’-TCCTTGTAGATCTCCTGCAG-3’
β-ACTIN	5’-TGGCACCCAGCACAATGAA-3’	5’-CTAAGTCATAGTCCGCCTAGAGCA-3’

Runx2: Runt-related transcription factor 2; Col-I: collagen type I; ALP: alkaline phosphatase; LPL: lipoprotein lipase; PPAR-γ: Peroxisome proliferator-activated receptor.

Primer sequences of osteogenesis related gene including Runx2, Col- I and ALP were used for RT-PCR to testy osteogenic differentiation. And primer sequences of adipogenesis related gene including LPL and PPAR-γ were used for RT-PCR to testy adipogenic differentiation.

Supplementary Table 2. Conditions of donators of hABMMSCs.

Patients	Smoking amount (cigarettes/ day)	Smoking history (year)	Bone type	Bone position

N1	—	—	Ⅲ	47
N2	—	—	Ⅲ	46
N3	—	—	Ⅱ	46
S1	20	>40	Ⅲ	36
S2	20	10-20	Ⅲ	37
S3	10	10-20	Ⅱ	46

hABMMSCs: human alveolar bone marrow mesenchymal stem cells; N1: non-smoking patient NO.1; N2: non-smoking patient NO.2; N3: non-smoking patient NO.3; N: the mean value of non-smoking patient; S1: smoking patient NO.1; S2: smoking patient NO.2; S3: smoking patient NO.3; S: the mean value of smoking patient.

Individual information including smoking and bone situation were collected from both nonsmoking and smoking patient. Compared with nonsmoking patient, the smoking amount (more than 10 cigarettes/ day) and history (longer than 10 years) of smoking patient reached the heavy smoking level.
